# Predictors of Acute Kidney Injury Resolution and Associated Clinical Outcomes Among Hospitalized Patients with Cirrhosis

**DOI:** 10.3390/jcm13216377

**Published:** 2024-10-24

**Authors:** Yazan Abboud, Anjana Rajan, Russell E. Rosenblatt, Clara Tow, Arun Jesudian, Brett E. Fortune, Kaveh Hajifathalian

**Affiliations:** 1 Department of Internal Medicine, Rutgers New Jersey Medical School, Newark, NJ 07103, USA; 2 Department of Internal Medicine, School of Medicine, Northwestern University Feinberg, Chicago, IL 60611, USA; anjanasitarajan@gmail.com; 3 Department of Gastroenterology and Hepatology, Weill Cornell Medicine, New York, NY 10065, USA; rur9017@med.cornell.edu (R.E.R.); abj9004@med.cornell.edu (A.J.); 4 Montefiore Einstein Center for Transplantation, Division of Hepatology, Montefiore Medical Center, The University Hospital for Albert Einstein College of Medicine, Bronx, NY 10467, USA; ctow@montefiore.org (C.T.); bfortune@montefiore.org (B.E.F.); 5 Department of Gastroenterology and Hepatology, Rutgers New Jersey Medical School, Newark, NJ 07103, USA

**Keywords:** acute kidney injury, cirrhosis, portal hypertension, hepatorenal syndrome, outcomes

## Abstract

**Background:** Acute kidney injury (AKI) is one of the common complications of liver cirrhosis. It occurs in nearly 20% of patients with cirrhosis who are hospitalized. Prior literature demonstrated that the AKI occurrence in patients with cirrhosis is independently associated with higher mortality. However, there are data assessing predictors and outcomes of AKI resolution in hospitalized patients with cirrhosis. Therefore, the aim of the current study was to identify clinical predictors of AKI resolution among inpatients with cirrhosis that are easily obtained and to evaluate the clinical outcomes of those patients. **Methods:** The current study is a retrospective cohort of patients with cirrhosis who were hospitalized and had AKI between 2012 and 2020 at a tertiary referral center. Patients included in this study were identified using the International Classification of Diseases 9 codes and then they were manually verified by two independent chart reviewers. AKI was classified according to the AKI Network (AKIN) serum creatinine (Cr) criteria, with AKIN resolution defined as AKIN stage 1 or lower at the time of discharge, while unresolved AKIN was defined as AKIN stage 2 or 3 at the time of discharge. For univariate analysis, Fisher’s exact and the two-sample T-test were utilized. For multivariable analysis, stepwise logistic regression was performed to evaluate variables associated with AKIN resolution. Survival curves were estimated and compared using the Kaplan–Meier method and Log-Rank Test. A *p*-value cutoff of 0.05 was used for statistical significance. **Results:** Between 2012 and 2020, there were 140 patients who were included (59% males). The majority of patients had viral hepatitis (54%) as the cirrhosis etiology with 80% of them having hepatitis C virus. Most patients had fluid-responsive AKI (49%), and stage 1 AKIN (69%). In terms of outcomes, the majority of patients (117 patients; 84%) had AKIN resolution at the time of discharge. In the multivariable analysis, after adjusting for clinical meaningful variables, our study shows that higher albumin value at the time of admission (adjusted Odds Ratio “aOR” = 3.28; *p* = 0.01) and non-metabolic dysfunction-associated steatotic liver disease (non-MASLD) cirrhosis (aOR = 9.43; *p* < 0.01) were variables associated with higher odds of AKIN resolution at the time of discharge. Conversely, we show that a higher Cr value at the time of admission was associated with lower odds of AKIN resolution at the time of discharge (aOR = 0.31; *p* < 0.01). When evaluating mortality, patients with unresolved AKIN at the time of discharge had higher rates of in-hospital mortality (*p* < 0.01) compared to those with resolved AKIN. Survival curve analyses using the Kaplan–Meier method indicated that patients with resolved AKIN experienced higher 90-day survival rates (*p* < 0.01). Additionally, those with resolved AKIN demonstrated greater transplant-free survival compared to patients with unresolved AKIN at both the 1-year (*p* = 0.04) and 3-year (*p* < 0.01) follow-ups. **Conclusions:** When evaluating clinical predictors of AKIN resolution in admitted patients with cirrhosis, our study showed that a higher admission albumin value and non-MASLD etiology of cirrhosis were associated with higher odds of AKIN resolution at the time of discharge. Conversely, a higher admission Cr value was associated with lower odds of AKIN resolution at the time of discharge. We also demonstrate that AKIN resolution during index admission was associated with improved short- and long-term transplant-free survival (up to 3 years). Our findings warrant external validation in larger cohorts to further evaluate the impact of inpatient AKI resolution on cirrhosis outcomes. Our findings can help clinicians predict AKIN outcomes and encourage more aggressive management of AKI, especially in high-risk patients, which can improve mortality.

## 1. Introduction

Acute kidney injury (AKI) is a severe complication of cirrhosis that occurs in approximately 20% of hospitalized patients with cirrhosis [1]. The American Gastroenterology Association (AGA) defines AKI as an abrupt increase in serum Creatinine (Cr) by ≥0.3 mg/dL within 48 h, or by an increase that is ≥50% from baseline Cr, or if the urine output is reduced below 0.5 mL/kg/h for more than six hours [2]. AKI can occur in patients with cirrhosis secondary to renal hypoperfusion (which represents the most common cause of AKI in patients with cirrhosis), hepatorenal syndrome (HRS), acute tubular necrosis (ATN), or post-renal causes such as urinary obstruction [1,3,4].

Previous studies have established that AKI in cirrhosis can be an independent predictor of mortality with an in-hospital death rate of 26% [5]. Prior data have also shown that the mortality rate of patients hospitalized with cirrhosis increases with the more advanced AKI stage [6,7]. Furthermore, the progression of AKI, based on the AKI Network (AKIN) criterion, during hospitalization magnifies the death risk independent of the AKIN stage at the time of admission [5]. Thus, early recognition and prevention of AKIN progression are critical for providers to halt this ominous process.

Given the increased risk of mortality associated with AKI in hospitalized patients with cirrhosis, researchers focused on investigating any potential risk factors that can predict AKIN progression in this population. Prior studies showed that urinary biomarkers have a role in predicting AKIN progression in hospitalized patients with cirrhosis and their associated mortality [8,9]. A multicenter prospective study was conducted in the US between 2009 and 2011 on 188 hospitalized patients with cirrhosis and showed that several urinary biomarkers including neutrophil gelatinase-associated lipocalin, interleukin-18 (IL-18), kidney injury molecule-1 [KIM-1], liver-type fatty acid-binding protein [L-FABP], and albuminuria, were independently associated with AKI progression and mortality [8]. Though promising, these biomarkers are currently not clinically feasible, especially in non-academic centers, due to cost, time constraints, and lack of availability, especially in non-academic centers. Therefore, there is an essential need to identify clinical predictors of AKIN progression in hospitalized patients with cirrhosis that are readily accessible.

While a growing body of literature has shown that AKI in patients with cirrhosis is associated with worse outcomes, there remains a lack of literature evaluating the prognosis of AKI resolution in hospitalized patients with cirrhosis [5]. Investigating these outcomes is essential, especially given the hypothesis that AKI resolution during index admission may lead to improvement in the outcomes of this population. In addition, data are scarce regarding short- and long-term outcomes of patients with cirrhosis who experienced AKI during their hospitalization.

Therefore, the primary aim of our study was to identify easily obtained clinical predictors of AKIN resolution in patients who are hospitalized and have cirrhosis. The secondary aims were to assess the associated short- and long-term clinical outcomes of those patients.

## 2. Methods

The current study is a retrospective single-center cohort study of hospitalized patients with cirrhosis and AKI between 2012 and 2020 at a tertiary referral center. The research protocol was reviewed and this study was approved by the Institutional Review Board Committee (IRB protocol #805019218) and was conducted in accordance with the ethical principles documented in the Declaration of Helsinki.

### 2.1. Inclusion and Exclusion Criteria

The inclusion criteria for our study were adult patients with cirrhosis aged ≥18 years old with evidence of AKI either at admission or during hospital stay. The exclusion criteria included patients with kidney or liver transplantation, pregnant patients, those on hemodialysis at the time of index admission, or patients with advanced chronic kidney disease who had a baseline serum Cr > 4 mg/dL.

### 2.2. Definitions and Included Variables

Eligible patients with liver cirrhosis were initially identified using the International Classification of Disease 9 (ICD-9) codes. Thereafter, the identified patients were manually verified by two independent chart reviewers who used liver biopsy or a combination of imaging, clinical, and laboratory findings to diagnose cirrhosis and verify patients’ eligibility. AKI was categorized using the AKIN serum creatinine criteria given its sensitivity, specificity, and ability to predict outcomes in hospitalized patients with AKI [10,11]. Baseline Cr was defined as the most recent stable Cr measurement within 365 days of index admission. When prior laboratory data were unavailable, a post-discharge Cr value was used as the baseline if the AKI episode had resolved and if the patient maintained a stable Cr value in follow-up laboratory data. AKIN resolution was defined as AKIN stage 1 or lower at the time of discharge. Unresolved AKIN was defined as AKIN stage 2 or 3 at the time of discharge. Based on the Kidney Disease Improving Global Outcomes (KDIGO) guidelines criteria, AKI resolution is defined by the absence of AKI criteria which is characterized by the decrease in serum Cr to below the threshold for AKI diagnosis as well as the improvement of urine output to baseline levels [12]. Since it is clinically challenging to maintain strict intake and output for all hospitalized patients to measure their urine output, we opted to choose the return of serum Cr to baseline levels as our definition for AKI resolution. Furthermore, based on the AKIN criteria, while stage 1 AKI corresponds to the risk class, stage 2 AKI corresponds to the injury class and stage 3 AKI corresponds to the failure class or patients requiring renal replacement therapy [11]. Prior studies have shown that patients’ outcomes in AKI stage 1 are generally better than in AKI stages 2 and 3, including those with cirrhosis [6,7,13]. This prompted the aforementioned definitions of resolved and unresolved AKI.

Demographic data and past medical history were determined based on documentation in charts. Discharge data were within 48 h of leaving the hospital. Collected variables included demographics, vital signs, co-morbidities, cirrhosis etiologies, AKI etiologies, laboratory values (including complete blood count, comprehensive metabolic panel, coagulation studies), admission medications, in-hospital complications, readmission data, and mortality.

### 2.3. Statistical Analysis

Categorical variables were reported as raw numbers and percentages, while continuous variables were reported as means with standard deviations. The Shapiro–Wilk test was conducted to evaluate whether the distribution of the data was normal. The chi-squared test, Fisher’s exact test, and the two- *t*-test were used for univariable analysis. Stepwise logistic regression was conducted to predict the variables associated with AKIN resolution with selection criteria of *p*-value cutoff at 0.05. Survival curves based on AKIN resolution status were estimated and compared using the Kaplan–Meier and Log-Rank tests.

## 3. Results

### 3.1. Demographics

There were 140 patients with cirrhosis and AKI who met inclusion criteria with available discharge data (Table 1). The average age was 66 years (±12) with male predominance (59%). Viral hepatitis was the most common cirrhosis etiology (54%) with 80% of those having hepatitis C virus (HCV) cirrhosis, followed by alcohol-associated liver disease (17%) and metabolic dysfunction-associated steatotic liver disease (MASLD) (16%). Fluid-responsive AKI (pre-renal AKI) was the most common AKI etiology (49%), with an average Cr of 2.17 (±1.26) in the entire cohort. Stage 1 AKIN was the most common AKI stage at presentation (69%). The average Child–Turcotte–Pugh (CTP) score was 8 (±2) with 52% of the cohort in class B (Table 1). The average MELD-Na score was 22 (±7).

### 3.2. AKIN Resolution Predictors

There were 117 patients with resolved AKIN (84%) and 23 patients without AKIN resolution (16%) upon discharge (Table 1). Baseline comorbidities were comparable between the two arms of this study except hypertension which was significantly more prevalent in patients with resolved AKIN (*p* = 0.011).

Univariate analysis showed that patients with AKIN resolution were more likely to have lower AKIN stage-at-presentation (*p* < 0.001), fluid-responsive AKI (*p =* 0.005), higher albumin (*p* = 0.001), lower Cr (*p* < 0.001) and lower CTP score (*p =* 0.039).

On multivariable stepwise logistic regression analysis, after adjusting for numerous demographic, clinical, and laboratory variables, we show that higher albumin at admission (aOR = 3.28, *p =* 0.01) and non-MASLD cirrhosis etiology (aOR = 9.43, *p =* 0.003) were associated with higher odds of AKIN resolution (Table 2). On the other hand, higher Cr at admission was associated with lower odds of AKIN resolution (aOR = 0.31, *p* < 0.001).

### 3.3. Short- and Long-Term Outcomes

**Figure 1:**
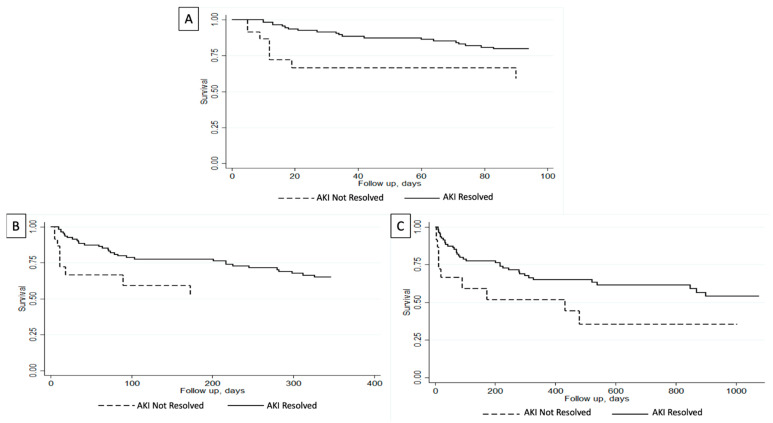
Survival analysis evaluating transplant-free survival for admitted patients with cirrhosis and acute kidney injury (AKI) based on the resolution of their AKI. (**A**) Greater 90-day transplant-free survival in patients with resolved AKI (*p* = 0.008). (**B**) Greater 1-year transplant-free survival in patients with resolved AKI (*p* = 0.04). (**C**) Greater 3-year transplant-free survival in patients with resolved AKI (*p* = 0.009) [14].

Short-term outcomes analysis in our cohort demonstrated that patients with unresolved AKIN were more likely to have been intubated (22% vs. 2%; *p* < 0.001) and had dialysis initiation (13% vs. 2%; *p* = 0.007) during index admission (Table 3). Furthermore, in-hospital mortality was noted in 4% of the entire cohort. Patients with unresolved AKIN had higher in-hospital mortality compared to those with resolved AKIN at the time of death (22% vs. 1%; *p* < 0.001). On the other hand, patients with resolved AKIN had a higher 30-day transplant-free survival (92% vs. 70%; *p =* 0.002) and a non-significant a higher 90-day transplant-free survival (82% vs. 65%; *p* = 0.061) (Table 3). Survival analysis using Kaplan–Meier curves showed that patients with resolved AKIN had a higher 90-day survival (*p* = 0.008) (Figure 1A).

When evaluating long-term outcomes analysis, three-year outcome data were available for 137 patients. Median follow-up was greater in patients with resolved AKIN (273 days vs. 20 days) and they had higher rates of being readmitted within 3 years (65% vs. 35%). Readmission reasons were all comparable between the two cohorts (Table 4). As for complication rates during readmissions, they were also comparable except that patients with unresolved AKIN upon first discharge had higher rates of undergoing hemodialysis during their readmission (67% vs. 0%; *p* < 0.001) (Table 5). Univariate analysis showed that patients with resolved AKIN had higher rates of transplant-free survival at 3-year follow-up (68% vs. 52%; *p =* 0.02). Furthermore, survival analysis using Kaplan–Meier curves showed that patients with resolved AKIN had higher overall transplant-free survival compared to those with unresolved AKIN at 1-year (*p =* 0.04) and 3-year (*p =* 0.009) follow-up (Figure 1B,C).

## 4. Discussion

When investigating predictors of AKIN resolution in hospitalized patients with cirrhosis, our study observed that higher albumin levels at admission and non-MASLD etiology of cirrhosis were associated with higher odds of AKIN resolution, whereas higher levels of Cr at admission were associated with lower odds of AKIN resolution, after adjusting for clinically meaningful variables. We also showed that AKIN resolution during index admission improved short- and long-term transplant-free survival (up to 3 years).

A systemic review and meta analysis of 30 studies including 18,474 hospitalized patients with cirrhosis showed that the incidence of AKI was 29% and that in-hospital mortality was six-fold higher in patients with AKI [15]. Some of the risk factors associated with developing AKI in hospitalized patients with cirrhosis include higher Child–Turcotte–Pugh score, ascites, and sepsis [15]. Despite the growing literature showing increasing morbidity and mortality in patients with cirrhosis hospitalized with AKIN, there remains no prior data evaluating the predictors of AKIN resolution in this ill population. Our study adds to the literature by identifying clinical and laboratory variables that can predict AKIN resolution.

A growing body of literature has been showing that higher albumin is associated with better outcomes in patients with AKI [16,17]. For instance, a large retrospective study including 740 hospitalized patients with AKI showed a negative correlation between albumin levels and mortality up to 1 year of follow-up [16]. This was thought to be in part due to the anti-inflammatory and antioxidant properties of albumin, along with its role in maintaining oncotic pressure and improving renal perfusion. This made it part of the management guidelines for patients with HRS [18]. Our study adds to the literature and shows that higher albumin levels were associated with higher odds of AKIN resolution in hospitalized patients with cirrhosis. In addition to the impact of albumin on kidney function, higher Cr at the time of admission has been noted to be associated with persistent kidney injury and worse outcomes including higher mortality [19]. The literature is scarce in evaluating the impact of admission Cr on AKI resolution in hospitalized patients with cirrhosis. Our study provides evidence showing that higher admission Cr is associated with lower odds of AKIN resolution in this population. Prior literature showed that patients with higher Cr tend to have reduced renal reserve which makes them more susceptible to persistent kidney injury [20]. This was demonstrated when evaluating AKI in most patients and not only in patients with cirrhosis. Furthermore, when evaluating cirrhosis related etiologies of AKI such as hepatorenal syndrome, the severe splanchnic vasodilation along with renal vasoconstriction and systemic circulatory changes due to advanced liver disease can lead to impaired renal function and potentially reduced reserve [21,22]. These changes in renal function in patients with AKI likely explain our findings showing that higher Cr at the time of admission is likely associated with persistent impaired renal function.

The role of hepatic factors, also known as hepatokines, in the pathophysiology of MASLD and its effect on kidney function has been studied [23]. Prior data suggested that hepatic factors such as Angiopoietin-Like Protein 4 (ANGPTL4), Angiopoietin-Like Protein 6, FGF21 (Fibroblast-growth factor 21), and Insulin-Like Growth Factor (IGF-1) may play a role in preventing AKI [24]. Given that these factors are expressed differently in MASLD patients, this can lead to metabolic dysfunction and increase AKI risk in patients with MASLD cirrhosis. This may explain the findings in our study of worse AKIN outcomes in MASLD patients compared to other cirrhosis etiologies. In addition, patients with MASLD tend to have a high prevalence of metabolic and cardiovascular risk factors such as obesity, hyperlipidemia, diabetes, chronic kidney disease, atherosclerosis, and hypertension [25,26,27]. These risk factors can affect renal function and are also likely to contribute to the worse outcomes of AKIN resolution in this population which was demonstrated in our study. Future studies are warranted to further investigate the role of hepatic factors in preventing AKI and possibly target these pathways in MASLD.

A systemic review and meta analysis of 32 studies showed that AKI in patients with cirrhosis was associated with higher odds of in-hospital mortality and also up to 1-year follow-up compared to patients without AKI [28]. Our study builds upon the existing literature by providing data showing that AKIN resolution in hospitalized patients with cirrhosis was associated with a greater transplant-free survival at 90-day, 1-year, and 3-year follow-ups. These findings highlight the importance of optimizing kidney function in hospitalized patients with as it can lead to improved mortality up to 3 years follow up.

Since the introduction of directly acting antiviral agent (DAA) regimens in the management of HCV, a growing literature demonstrated their favorable outcomes on kidney function [29]. A study by Coppola et al. evaluating 403 patients with HCV demonstrated that DAA correlated with improvement in kidney function [30]. While our study does not have data regarding whether patients with HCV were receiving DAA, it is reasonable to hypothesize that the majority of these patients were either treated or being treated and that could potentially influence the outcomes, leading to higher rates of AKIN resolution. However, the proportion of patients with HCV in our study did not differ between the two arms (resolved AKIN 88% vs. non-resolved AKIN 77%; *p =* 0.32) and thus, while DAA could potentially impact the outcomes and lead to AKIN resolution, their effect is likely to be similar on both study arms.

Our study highlights several clinical variables that can guide clinicians to predict which group of patients are at higher odds of AKI resolution and to identify patients at risk of persistent AKI. This is essential as our findings demonstrate that patients who had AKI resolution had better short- and long-term survival. Clinicians should aggressively manage AKI in hospitalized patients with cirrhosis especially in patients with MASLD-cirrhosis, higher Cr at admission, and low Albumin at admission since they are at risk of persistent AKI upon discharge. Multidisciplinary approach with the nephrology team might be warranted to comprehensively evaluate and manage those patients with the goal of resolving their AKI which may lead to improved mortality.

Some of the strengths of our study include the granularity of data evidenced by the available demographics, disease characteristics, and clinical and laboratory variables for the population included in our study. We also utilized multivariable logistic regression for the analysis while adjusting for numerous clinically meaningful variables. Furthermore, we provide novel findings demonstrating predictors of AKIN resolution in hospitalized patients with cirrhosis and showing improved long-term mortality in those with resolved AKIN up to follow-up to 3 years. However, our study suffers from several limitations which include its small sample size, the inherent limitations due to its retrospective design, and the longer in median follow-up period in patients with AKI resolution compared to the cohort with persistent AKI. The difference in median follow-up between the cohorts could explain why patients who experienced AKIN resolution had higher readmission rates at 3-year follow-up compared to those with persistent AKIN. Another explanation is the higher in-patient mortality in patients with unresolved AKIN. It is also notable that the median CTP and MELD-Na scores in our study were high which can lead to higher re-admission rates. While this can also potentially explain the high readmission rates in patients with AKIN resolution, CTP score was significantly higher in patients with unresolved AKIN and MELD-Na score was non-significantly higher in patients with unresolved AKIN as well. Thus, it is less likely that this solely explains the higher readmission rates of patients with resolved AKIN [31]. Furthermore, some participants had missing data on some of their demographics, disease characteristics, and outcomes. The missing data include the usage of Octreotide or Terlipressin which can improve AKI especially hepatorenal syndrome. The presence of CKD and HCC in part of our sample size can influence the outcomes of this study. To account for this possible influence, we did not include patients with end-stage renal disease who were undergoing dialysis at the time of admission. We defined AKI on CKD by the risk of serum Cr compared to their baseline Cr prior to admission if data were available. We also adjusted for the status of CKD in our multivariable regression model. As for HCC, it can affect outcomes, especially if patients are receiving chemotherapy that might lead to nephrotoxicity. However, in our multivariable regression model, we also adjusted for the status of HCC when evaluating predictors of AKI resolution. Another limitation of our study includes identifying the Cr baseline for patients without prior laboratory results by a post-discharge Cr value if the AKI had resolved and the Cr was stable. This method was chosen given the retrospective nature of our study and the lack of available data on all the patients. While this can influence results, we did not include post-discharge Cr data on patients whose AKI did not resolve or had unstable Cr values upon discharge. Lastly, due to the overlapping clinical features of hepatorenal syndrome and acute tubular necrosis and the potential co-existence of both etiologies in the population of our study, we grouped them into one category. Future studies should investigate AKI resolution predictors and outcomes based on the presence of HRS.

## 5. Conclusions

In conclusion, we provide clinical and easy-to-obtain predictors of AKIN progression and resolution in hospitalized patients with cirrhosis. Our study demonstrates that higher albumin at admission and non-MASLD cirrhosis etiology were associated with higher odds of AKIN resolution, whereas higher Cr at admission was associated with lower odds of AKIN resolution. We also provide novel data showing that AKIN resolution in index admission improves short- and long-term transplant-free survival up to 3 years follow-up. Our findings warrant external validation in larger cohorts to further evaluate the impact of inpatient AKI resolution on cirrhosis outcomes. This could aid clinicians in predicting AKIN outcomes and encourage aggressive AKI management in high-risk patients, thus reducing mortality.

## Figures and Tables

**Table 1:** Patient demographics and hospital characteristics at admission of admitted patients with cirrhosis with acute kidney injury based on AKIN resolution status.

	Total Cohort N = 140	AKIN Resolved (Discharge AKIN 0 or 1) N = 117	AKIN Unresolved (Discharge AKIN 2 or 3) N = 23	*p*-Value
Age in years—mean ± SD	66 ± 12	66 ± 12	64 ± 14	0.41
Male—n (%)	83/140 (59%)	73/117 (62%)	10/23 (43%)	0.09
Cirrhosis etiology—n (%)				0.62
Alcohol	23/138 (17%)	21/115 (18%)	2/23 (8%)	
MASLD/MASH	22/138 (16%)	16/115 (14%)	6/23 (26%)	
Viral hepatitis	74/138 (54%)	56/115 (49%)	13/23 (57%)	
HBV	20/74 (27%)	15/56 (27%)	5/13 (38%)	0.46
HCV	59/74 (80%)	49/56 (88%)	10/13 (77%)	0.32
Hx of CVD—n (%)	48/128 (38%)	43/108 (40%)	5/20 (25%)	0.20
Hx of HTN—n (%)	93/140 (66%)	83/117 (71%)	10/23 (43%)	0.011
Hx of HCC—n (%)	33/140 (24%)	25/117 (21%)	8/23 (35%)	0.16
Hx of CKD—n (%)	56/140 (40%)	46/117 (39%)	10/23 (43%)	0.71
Temperature (C deg)	36.6 ± 0.4	36.6 ± 0.4	36.6 ± 0.3	0.76
HR (bpm)	81 ± 18	81 ± 19	86 ± 17	0.22
Systolic BP (mm Hg)	125 ± 27	124 ± 26	127 ± 27	0.61
Diastolic BP (mm Hg)	72 ± 14	71 ± 13	74 ± 15	0.38
MAP (mm Hg)	90 ± 17	89 ± 16	92 ± 18	0.45
AKIN stage at diagnosis—n (%)				<0.001
Stage 1	96/140 (69%)	87/117 (74%)	9/23 (39%)	
Stage 2	27/140 (19%)	23/117 (20%)	4/23 (17%)	
Stage 3	17/140 (12%)	7/117 (6%)	10/23 (43%)	
Cr—mean ± SD	2.17 ± 1.26	2.0 ± 0.9	3.3 ± 2.2	<0.001
Na—mean ± SD	134 ± 6	134 ± 6	134 ± 7	0.93
Bilirubin—mean ± SD	3.19 ± 4.63	3.1 ± 4.8	3.6 ± 4.2	0.63
INR—mean ± SD	1.7 ± 1.6	1.8 ± 1.7	1.5 ± 0.5	0.45
BUN—mean ± SD	41 ± 24	40 ± 22	47 ± 30	0.17
Albumin—mean ± SD	2.8 ± 0.8	2.9 ± 0.8	2.3 ± 0.8	0.001
WBC—mean ± SD	7.9 ± 4.3	7.6 ± 4.0	8.9 ± 5.5	0.19
Hgb—mean ± SD	10.9 ± 2.4	10.9 ± 2.5	10.6 ± 2.1	0.54
Platelets—mean ± SD	148 ± 100	145 ± 97	165 ± 121	0.39
Lactate—mean ± SD	2.12 ± 1.54	2.1 ± 1.6	2.1 ± 1.1	0.98
AST—mean ± SD	88 ± 147	82 ± 130	116 ± 223	0.32
ALT—mean ± SD	56 ± 96	54 ± 97	61 ± 85	0.76
AKI etiology—n (%)				0.005
Fluid responsive (pre-renal)	73/139 (49%)	68/116 (59%)	5/23 (22%)	
Fluid unresponsive (ATN + HRS)	27/139 (19%)	19/116 (16%)	8/23 (35%)	
Other/Unknown	39/139 (28%)	29/116 (25%)	10/23 (43%)	
Ascites at admission—n (%)				0.55
Slight	27/139 (19%)	23/116 (20%)	4/23 (17%)	
Moderate/Large	47/139 (34%)	37/116 (32%)	10/23 (43%)	
CTP class at admission				0.053
A	27/130 (21%)	24/110 (22%)	3/20 (15%)	
B	67/130 (52%)	60/110 (55%)	7/20 (35%)	
C	36/130 (28%)	26/110 (24%)	10/20 (50%)	
CTP score—mean ± SD	8 ± 2	8.2 ± 2.1	9.4 ± 2.4	0.039
Na-Meld score—mean ± SD	22 ±7	21 ± 7	24 ± 6	0.09
Admission medication—n (%) #				
Beta-blockers	81/140 (58%)	68/117 (58%)	13/23 (57%)	0.88
Statin	33/140 (24%)	29/117 (25%)	4/23 (17%)	0.44
PPI	65/140 (46%)	57/117 (49%)	8/23 (35%)	0.22
NSAID	2/140 (1%)	2/117 (2%)	0/23 (0%)	0.52
Aspirin	28/140 (20%)	25/117 (21%)	3/23 (13%)	0.36
Lactulose	42/140 (30%)	34/117 (29%)	8/23 (35%)	0.58
Rifaximin	38/140 (27%)	33/117 (28%)	5/23 (22%)	0.52

AKIN: Acute Kidney Injury Network; MASLD: metabolic dysfunction-associated steatotic liver disease; MASH: metabolic dysfunction-associated steatohepatitis; CVD: cardiovascular disease; HTN: hypertension; HCC: hepatocellular carcinoma; CKD: chronic kidney disease; HR: heart rate; BP: blood pressure; MAP: mean arterial pressure; ATN: acute tubular necrosis; HRS: hepatorenal syndrome; CTP: Child–Turcotte–Pugh; PPI: proton pump inhibitors; HBV: hepatitis B virus; HCV: hepatitis C virus. # The medications listed were those that patients were taking at the time of admission. These medications may not have been fully resumed during their hospital stay, depending on their indications, side effects, and the clinical status of each patient.

**Table 2:** Multivariable analysis for variables associated with AKIN resolution after adjusting for demographic, clinical, and laboratory data.

Variable	Adjusted Odds Ratio (aOR)	95% CI	*p*-Value
Albumin at Admission	3.28	1.25–8.56	0.01
Creatinine at Admission	0.31	0.17–0.59	<0.001
Non-MASLD/MASH Cirrhosis Etiology	9.43	6.02–11.07	0.003

Variables included in the multivariable analysis: gender, age, AKIN stage-at-diagnosis, AKIN etiology, HTN, CVD, HCC, cirrhosis etiology (MASLD/MASH vs. non-MASLD/MASH), ICU admission, dialysis during admission, infection during admission, length of stay, heart rate at admission, systolic, diastolic, and MAP pressures at admission, MELD-Na at admission or CTP at admission, sodium at admission, BUN at admission, creatinine at admission, albumin at admission, total bilirubin at admission, and INR at admission. AKIN: Acute Kidney Injury Network; MASLD: metabolic dysfunction-associated steatotic liver disease; MASH: metabolic dysfunction-associated steatohepatitis; CVD: cardiovascular disease; HTN: hypertension; HCC: hepatocellular carcinoma; HR: heart rate; BP: blood pressure; MAP: mean arterial pressure; ATN: acute tubular necrosis; HRS: hepatorenal syndrome; CTP: Child–Turcotte–Pugh.

**Table 3:** Outcomes of admitted patients with cirrhosis with acute kidney injury based on AKIN resolution status.

	Total Cohort N = 140	AKIN Resolved (Discharge AKIN 0 or 1) N = 117	AKIN Unresolved (Discharge AKIN 2 or 3) N = 23	*p*-Value
Bleeding in hospital—n (%)	17/140 (12%)	15/117 (13%)	2/23 (9%)	0.58
Infection in hospital—n (%)	43/140 (31%)	35/117 (30%)	8/23 (35%)	0.64
ICU during hospital stay—n (%)	25/140 (18%)	19/117 (16%)	6/23 (26%)	0.26
Dialysis initiation—n (%) ^	5/140 (4%)	2/117 (2%)	3/23 (13%)	0.007
Intubation in hospital—n (%)	7/140 (5%)	2/117 (2%)	5/23 (22%)	<0.001
Length of stay—days	11 ± 9	12 ± 11	9 ± 6	0.26
Mean follow-up time—days	140 ± 606	578 ± 632	214 ± 306	0.008
In-hospital mortality—n (%)	6/140 (4%)	1/117 (1%)	5/23 (22%)	<0.001
Transplant-free survival—n (%)	81/137 (59%)	69/114 (61%)	12/23 (52%)	0.45
90-day transplant-free survival—n (%)	109/137 (80%)	94/114 (82%)	15/23 (65%)	0.06
30-day transplant-free survival—n (%)	121/137 (88%)	105/114 (92%)	16/23 (70%)	0.002
Liver transplants—n (%)	14/140 (10%)	13/117 (11%)	1/23 (4%)	0.32

^ Dialysis was initiated if clinically indicated after multidisciplinary discussion between the primary, gastroenterology/hepatology, and nephrology teams. ICU: intensive care unit.

**Table 4:** Follow up and readmission data stratified by AKIN stage at discharge of index admission.

	Total Cohort N = 137	AKIN Resolved (Discharge AKIN 0 or 1) N = 114	AKIN Unresolved (Discharge AKIN 2 or 3) N = 23	*p*-Value
Median follow up time (days)		273	20	0.004
Readmission within 1 year—n (%)	73/137 (53%)	65/114 (57%)	8/23 (35%)	0.051
Readmission within 3 years—n (%)	82/137 (60%)	74/114 (65%)	8/23 (35%)	0.007
Number of readmissions—mean ± SD		0.65 ± 0.48	0.35 ± 0.49	0.007
Time to first readmission—days	142 ± 180	142 ± 187	145 ± 103	0.97
Reason for Readmission				
Liver-related readmission (HCC, HE, LFT, SBP, ascites, portal HTN bleed)	15/82 (18%)	13/74 (18%)	2/8 (25%)	0.61
HCC	1/82 (1%)	1/74 (1%)	0/8 (0%)	1
HE	4/82 (5%)	4/74 (5%)	0/8 (0%)	1
LFT	1/82 (1%)	0/74 (0%)	1/8 (13%)	0.09
SBP	1/82 (1%)	0/74 (0%)	1/8 (13%)	0.09
Ascites	7/82 (9%)	7/74 (9%)	0/8 (0%)	1
Portal HTN bleed	1/82 (1%)	1/74 (1%)	0/8 (0%)	1
Non-liver-related Readmission	67/82 (82%)	61/74 (82%)	6/8 (75%)	0.63
AKI	17/82 (21%)	13/74 (18%)	4/8 (50%)	0.053
Genitourinary	1/82 (1%)	1/74 (1%)	0/8 (0%)	1
Cardiac	11/82 (13%)	11/74 (15%)	0/8 (0%)	0.58
Hematology	2/82 (2%)	2/74 (3%)	0/8 (0%)	1
Metabolic	3/82 (4%)	3/74 (4%)	0/8 (0%)	1
Neurology	1/82 (1%)	0/74 (0%)	1/8 (13%)	0.09
Ophthalmology	1/82 (1%)	1/74 (1%)	0/8 (0%)	1
Orthopedics	4/82 (5%)	4/74 (4%)	0/8 (0%)	1
Psychiatry	1/82 (1%)	1/74 (1%)	0/8 (0%)	1
Pulmonology	2/82 (2%)	2/74 (3%)	0/8 (0%)	1
Non-liver gastroenterology	4/82 (5%)	4/74 (4%)	0/8 (0%)	1
Other gastrointestinal bleeding	1/82 (1%)	1/74 (1%)	0/8 (0%)	1
Infectious-related readmissions				
CLABSI	1/82 (1%)	1/74 (1%)	0/8 (0%)	1
UTI	4/82 (5%)	3/74 (4%)	1/8 (13%)	0.34
Bacteremia	1/82 (1%)	1/74 (1%)	0/8 (0%)	1
Cellulitis	3/82 (4%)	3/74 (4%)	0/8 (0%)	1
Osteomyelitis	1/82 (1%)	1/74 (1%)	0/8 (0%)	1
Other	8/82 (10%)	8/74 (11%)	0/8 (0%)	1
Unknown	1/82 (1%)	1/74 (1%)	0/8 (0%)	1

HCC: hepatocellular carcinoma, HE: hepatic encephalopathy, SBP: spontaneous bacterial peritonitis, HTN: hypertension, AKI: acute kidney injury, CLABSI: central line-associated bloodstream infection, and UTI: urinary tract infection.

**Table 5:** Long-term outcome data stratified by AKIN stage at discharge of index admission.

	Total Cohort N = 137	AKIN Resolved (Discharge AKIN 0 or 1) N = 114	AKIN Unresolved (Discharge AKIN 2 or 3) N = 23	*p*-Value
Complications During Admission				0.12
AKI	12/34 (35%)	10/28 (36%)	2/6 (33%)	1
Death	2/34 (6%)	2/28 (7%)	0/6 (0%)	1
LVP	2/34 (6%)	2/28 (7%)	0/6 (0%)	1
LVP, portal HTN bleed	1/34 (3%)	1/28 (4%)	0/6 (0%)	1
HE	1/34 (3%)	1/28 (4%)	0/6 (0%)	1
HD initiated	4/34 (12%)	0/28 (0%)	4/6 (67%)	0.0003
TIPS	1/34 (3%)	1/28 (4%)	0/6 (0%)	1
Liver transplant	1/34 (3%)	1/28 (4%)	0/6 (0%)	1
TIPS, ascites	1/34 (3%)	1/28 (4%)	0/6 (0%)	1
Amputation	1/34 (3%)	1/28 (4%)	0/6 (0%)	1
Ascites	1/34 (3%)	1/28 (4%)	0/6 (0%)	1
Metabolic	3/34 (9%)	3/28 (11%)	0/6 (0%)	1
Infectious complications				
Infection, ascites	1/34 (3%)	1/28 (4%)	0/6 (0%)	1
LVP, MRSA, UTI	1/34 (3%)	1/28 (4%)	0/6 (0%)	1
PNA	1/34 (3%)	1/28 (4%)	0/6 (0%)	1
UTI	1/34 (3%)	1/28 (4%)	0/6 (0%)	1
UTI, AKI	1/34 (3%)	1/28 (4%)	0/6 (0%)	1
Liver transplant after discharge—n (%)	13/137 (9%)	12/114 (11%)	8/23 (35%)	0.35
1-year transplant-free survival (%)	96/137 (70%)	82/114 (72%)	14/23 (61%)	0.06
3-year transplant-free survival (%)	89/137 (65%)	77/114 (68%)	12/23 (52%)	0.02

LVP: large volume paracentesis, HD: hemodialysis, TIPS: transhepatic intrajugular portosystemic shunt, MRSA: Methicillin-Resistant Staphylococcus Aureus, UTI: urinary tract infection, AKI: acute kidney injury, and PNA: pneumonia.

## Data Availability

The data used in this study are available upon request from the correspondence author.
